# Influence of Atmosphere and Temperature on Polycyclic Aromatic Hydrocarbon Emissions from Green Anode Paste Baking

**DOI:** 10.1021/acsomega.3c01411

**Published:** 2023-05-10

**Authors:** Kamilla Arnesen, Thor Anders Aarhaug, Kristian Etienne Einarsrud, Gabriella M. Tranell

**Affiliations:** † Department of Materials Science and Engineering, Norwegian University of Science and Technology (NTNU), 7034 Trondheim, Norway; ‡ SINTEF Industry, 7034 Trondheim, Norway

## Abstract

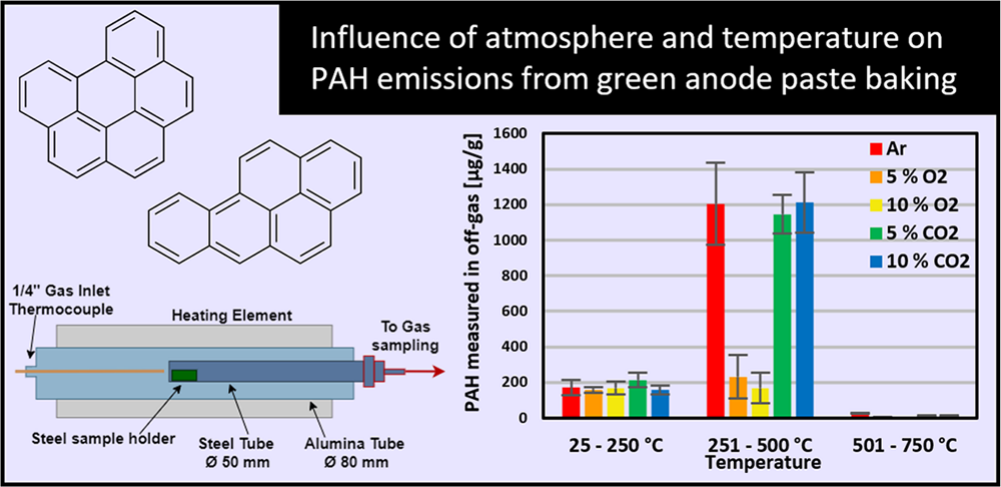

Coal tar pitch, a well-known source of polycyclic aromatic hydrocarbons (PAHs), is used as a binder of petroleum coke in prebaked anodes used for electrolysis of aluminum. Anodes are baked up to 1100 °C over a 20-day period, where flue gas containing PAHs and volatile organic compounds (VOCs) are treated using techniques such as regenerative thermal oxidation, quenching, and washing. Conditions during baking facilitate incomplete combustion of PAHs, and due to the various structures and properties of PAHs, the effect of temperature up to 750 °C and various atmospheres during pyrolysis and combustion were tested. PAH emissions from green anode paste (GAP) dominate in the temperature interval of 251–500 °C, where PAH species of 4–6 rings make up the majority of the emission profile. During pyrolysis in argon atmosphere, a total of 1645 μg EPA-16 PAHs are emitted per gram of GAP. Adding 5 and 10% CO_2_ to the inert atmosphere does not seem to affect the PAH emission level significantly, at 1547 and 1666 μg/g, respectively. When adding oxygen, concentrations decreased to 569 μg/g and 417 μg/g for 5% and 10% O_2_, respectively, corresponding to a 65% and 75% decrease in emission.

## Introduction

Polycyclic aromatic hydrocarbons (PAHs) are a diverse class of organic compounds, containing two or more fused aromatic rings. Many PAHs are classified as mutagenic, carcinogenic, and persistent organic pollutants, and reduced exposure and emissions are therefore recommended by the Council of the European Union.^1,2^ Prebaked carbon anodes for aluminum production are a mix of petroleum coke and coal tar pitch, baked in an anode baking furnace up to 1100 °C over a 20-day cycle. Coal tar pitch is a well-known source of PAHs. The pitch serves as a binder for the coke particles and as an energy source for the baking through combustion of volatile organic compounds (VOCs).^3^ In addition, minor elements including sulfur and trace elements such as vanadium, nickel, and iron will be present in the coke and pitch.^4^ Flue gas containing PAHs and VOCs from the baking furnace is often treated using regenerative thermal oxidation (RTO) technology, in addition to quenching and washing towers.^5^ In the research by Wittgens et al.,^6^ a pilot scale combustion chamber with optimized combustion control is presented to facilitate reduced PAH and tar emission in ferroalloy production, finding stoichiometry, gas flow, and temperature to be important conditions.

During anode baking the levels of oxygen in the flue gas have been measured to be between 3 and 11%.^7^ Anodes are also reported to be reactive with air and CO_2_ during the electrolysis process, at temperatures between 520 and 960 °C. This level of oxygen and thermal conditions may however not be enough to facilitate complete combustion of PAH. Chevarin et al.^8^ tested the O_2_ and CO_2_ reactivity of baked anodes and its constituents and found the coke to be most reactive with air, while both coke and anode butts showed high reactivity with CO_2_ at 960 °C. The coal tar pitch showed the lowest reactivity in all experiments, and this is thought to be influenced by the change in properties of the pitch when baked alone, and with coke particles.

The different structures of PAHs, linear, angular, and cluster, influence the reactivity of the molecules.^9^ Angular structures are more stable than linear, as studies show this to be due to better π-interactions, based on Clar’s model, and interactions between hydrogen atoms in the bay region.^10^ This region is found to increase the molecules’ mutagenic and carcinogenic activity.^11^ Another influencing factor for PAH stability is the number of rings. Larger PAH molecules have a greater resistance to degradation at ambient conditions, due to increased aromaticity. Degradation mechanisms of large PAH through thermal treatment and chemical oxidation can produce PAH of a smaller size as intermediates if enough energy is not supplied.^12^ Sun^13^ observed temperature as being a great influence on radical and PAH formation by way of intermolecular reorganization to increase molecular stability and aromaticity, at conditions where complete oxidation is not achieved. Liu et al.^14^ investigated PAH emissions from coal combustion in a fluidized bed combustor. In this study, incomplete combustion of PAHs was found at temperatures up to 900 °C, being strongly influenced by the level of excess air in the reactor. Pujro et al.^15^ investigated catalytic cracking of heavy aromatic molecules at 450 °C over a fluidized catalytic cracking zeolite catalyst with and without nickel and vanadium. The condensed polyaromatic compounds were found to have an increased activity with the catalysts over the thermal cracking, and this activity was found to increase with the number of aromatic rings.

The main objective of this study has been to investigate the effect of temperature and varying atmospheres on the EPA-16 PAH emissions from green anode paste (GAP) during initial heating of the anode baking process. Conditions at inert pyrolytic and oxidizing conditions were investigated in a controlled laboratory setup to better understand PAH emission profiles from green anode paste.

## Experimental Methodology

**Figure 1:**
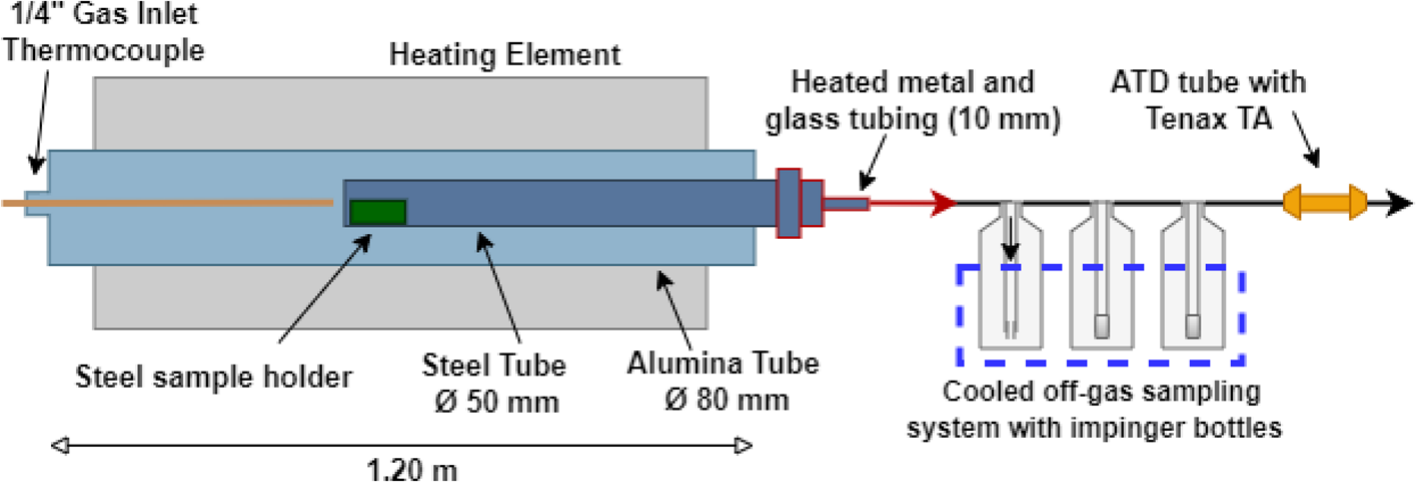
Sketch showing the alumina resistance furnace and off-gas sampling system used for experiments with green anode paste baking.

GAP for the experiments was supplied by an industrial aluminum production partner. The paste contains calcined petroleum coke, anode butts, and coal tar pitch, which was prepared by milling (Herzog Maschinenfabrik, Osnabrück, Germany). Representative sampling was performed following sample splitting and the spoon method, as described by Petersen et al.^16^ PAH composition in the off-gas was measured by performing experiments in a laboratory scale alumina tube resistance furnace (Nabertherm, RHTH 120-300/16-18) (Figure 1), where the off-gas was purged, and the organic content was collected in chilled 2-propanol (≥98% Technical, VWR Chemicals), using ice, and further analyzed by GC-MS (CP7462 Agilent column, Xtr EI 350 ion source). Samples were prepared by direct injection of internal standards containing deuterated PAH congeners and analyzed using selected ion monitoring (SIM).

**Figure 2:**
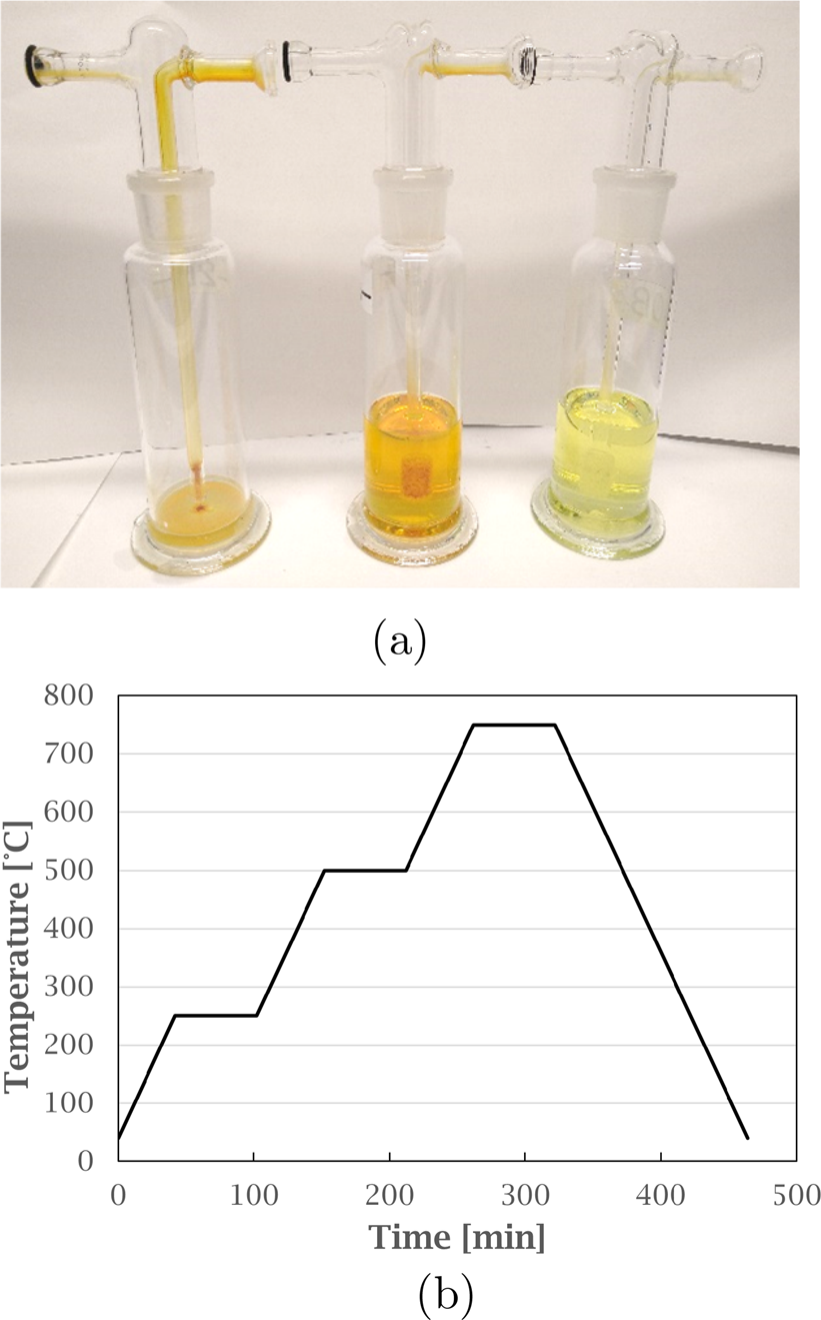
(a) Bottles with 2-propanol used for collecting PAH species from the off-gas. Bottles with impinger and frits (P0) are used. (b) Temperature ramping program used in the baking of GAP for PAH analysis.

For each experiment, 5.00 g GAP was placed in a steel holder and heated in a selected atmosphere with a set temperature ramping program (heating rate 5 °C/h), allowing off-gas sampling and exchange of bottles (Figure 2a) through three temperature intervals, 25–250, 251–500, and 501–750 °C (Figure 2b). The bottles were changed and ice refilled at the end of a hold period, while the gas stream was paused using a ball valve. This temperature range was chosen to study the evaporation of PAH from the raw material and the initial emission mechanism.

Different atmospheres were tested using a gas flow of 0.300 standard liters per minute. The atmosphere details are presented in Table 1. For experiments with different concentration of O_2_ and CO_2_, air (Technical grade, Linde Gas AS, Trondheim, Norway) and CO_2_ (≥99.7%) were diluted in argon (Instrument 5.0) to achieve the correct concentration levels.

**Table 1:** Atmospheres and Concentrations Tested for GAP Baking with PAH Analysis

atmosphere	concentration (%)
Argon	100
O_2_	5
O_2_	10
CO_2_	5
CO_2_	10

Experiments were performed in triplicates, except for 100% Ar, which was performed one additional time to test for breakthrough of PAH through the three sampling bottles. This test was performed by adding an analytical thermal desorption (ATD) tube filled with the absorbent Tenax TA and glass wool, to the sampling line after the last sampling bottle (Figure 1). The off-gas from the furnace will pass through the bubbling bottles and the ATD tube, where, if present, light PAH will absorb on Tenax TA. The glass wool is present to filter out water and any particles in the stream and shield the absorbent material. The PAH content in the sampling tube was analyzed using thermal desorption and GC-MS.

PAH content in the GAP material was also analyzed using pyrolysis GC-MS. Pyrolysis was performed using a 4 μg sample placed in a tandem μ-reactor (Frontier Lab 3050TR) for 1 min. Analysis was performed once at 400 and 600 °C, and thrice at 750 °C. Compounds were separated and detected using GC-MS (Agilent Technologies; GC 7890B and MSD 5977B), and identified by the NIST Library.

## Results and Discussion

### GAP Sample Weight Change

**Figure 3:**
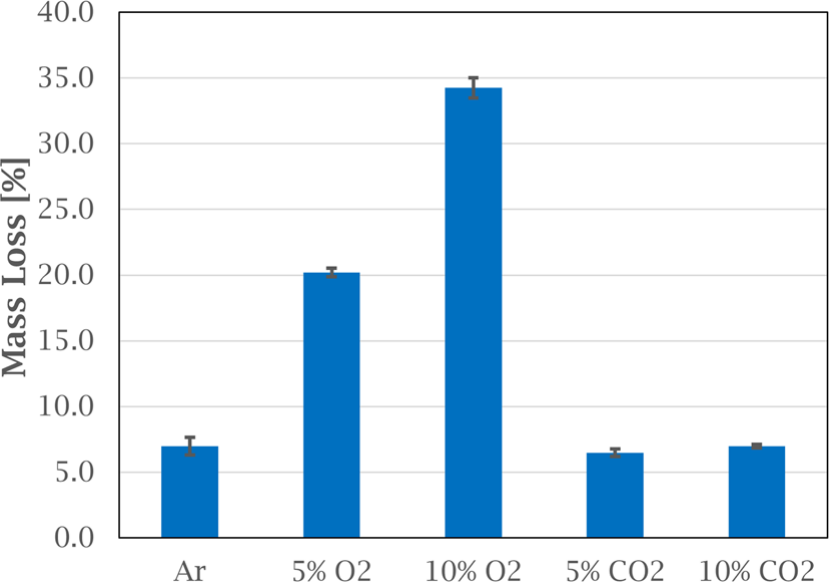
Average mass loss (%) for GAP samples at varying atmospheres. Error bars show the variation of triplicate experiments.

All GAP samples used to perform the PAH emissions tests were weighed before and after each experiment at room temperature to investigate sample mass loss. The average mass loss (%) is presented in Figure 3 for all atmospheres. Inert and CO_2_ atmosphere show a similar mass loss of 6.5–7.0%, independent of the concentration of CO_2_, which could be caused by the thermal evaporation of volatile organic components in the sample. The weight loss increased when oxygen was added, to 20.2% and 34.2% for 5% and 10% O_2_, respectively.

### Influence of Temperature

**Figure 4:**
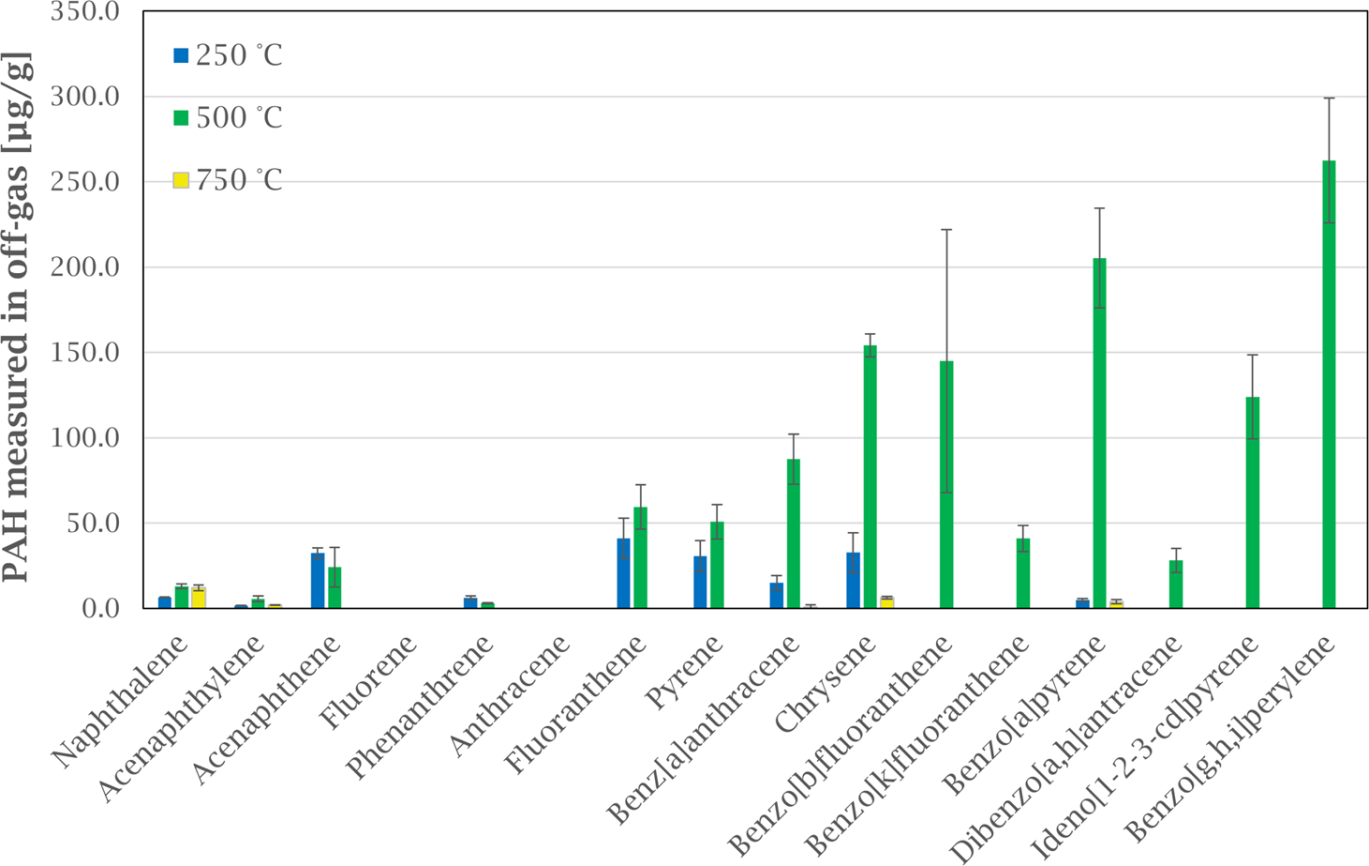
Off-gas emission of PAH species at various temperature intervals from heating green anode paste in argon atmosphere. Error bars show the standard deviation for each compound based on three experiments.

All experiments showed a PAH emission profile similar to what is shown in Figure 4, when comparing the effect of temperature. Emission is presented as μg EPA-16 PAH per gram of GAP from experiments in the alumina tube furnace.

Low molecular weight (LMW) PAHs (2 and 3 rings) are the main contributors to the emission at low temperatures, from room temperature to 250 °C. Up to 500 °C, the high molecular weight (HMW) PAHs (4–6 rings) are emitted, at a higher concentration level. Lastly, PAH emissions decrease significantly for temperatures between 500 and 750 °C. This observation fits with the general range of boiling points for PAH-16 compounds, starting at 218 °C for Naphthalene and reaching 550 °C for Benzo[g,h,i]perylene.^17^

**Figure 5:**
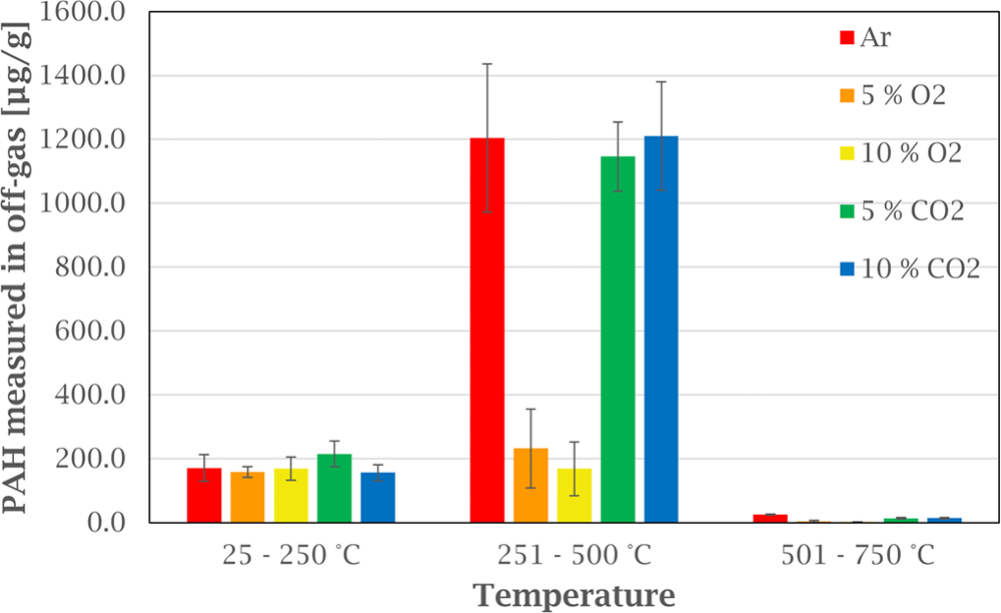
Off-gas emissions of EPA-16 PAHs at different temperature intervals from heating green anode paste in various atmospheres. Error bars show the standard deviation for the PAH concentration for each temperature interval based on three experiments.

This is also evident in Figure 5, where the total concentration of PAH from each temperature interval is presented.

Figure 5 also shows that the total PAH emissions level at low temperatures seem to be independent of the atmospheres tested, as the same level of emission within the standard deviation.

### Influence of Atmosphere

**Figure 6:**
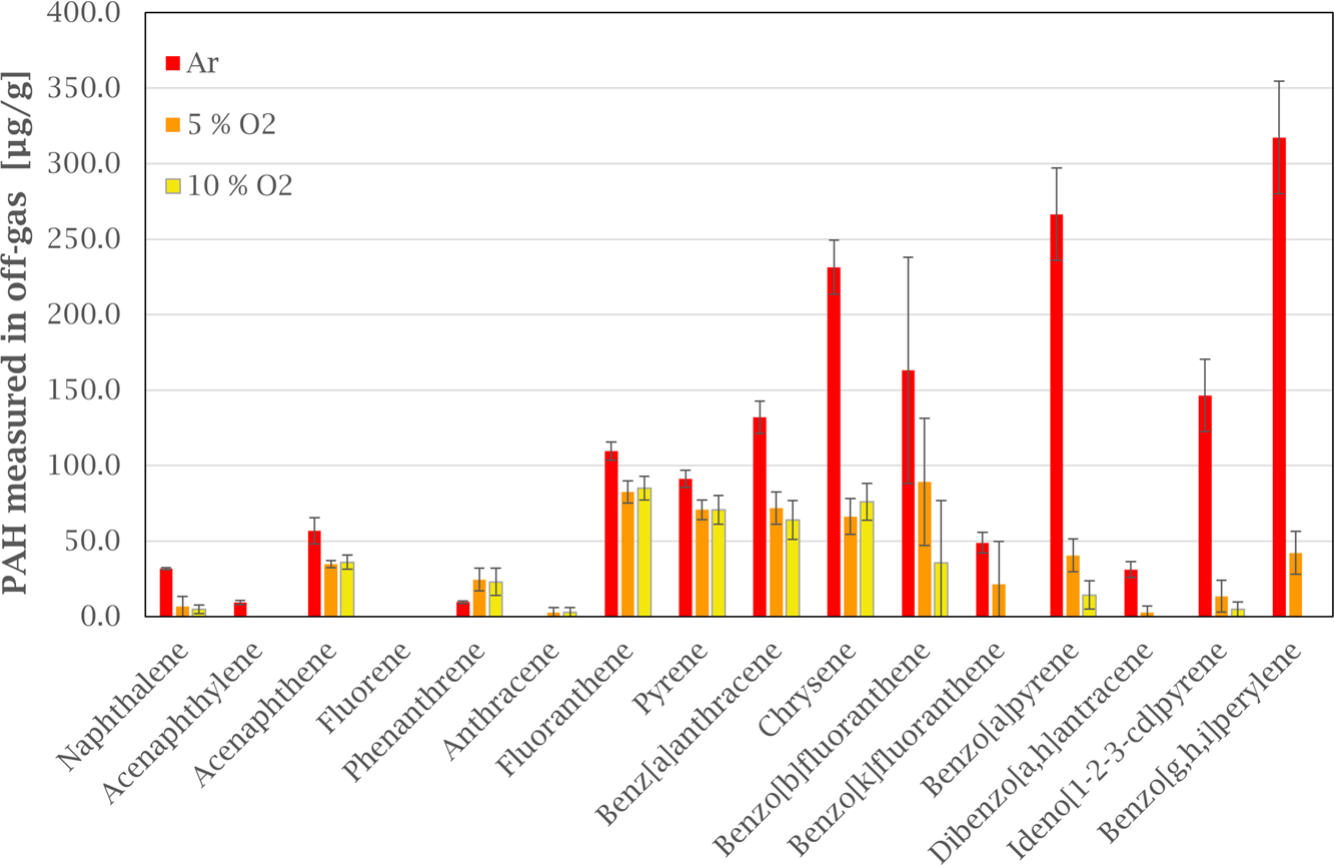
Comparing EPA-16 PAH emissions in off-gas from green anode paste heated in argon and oxygen (5 and 10%) atmospheres. Error bars show the standard deviation for each compound based on three experiments.

**Figure 7:**
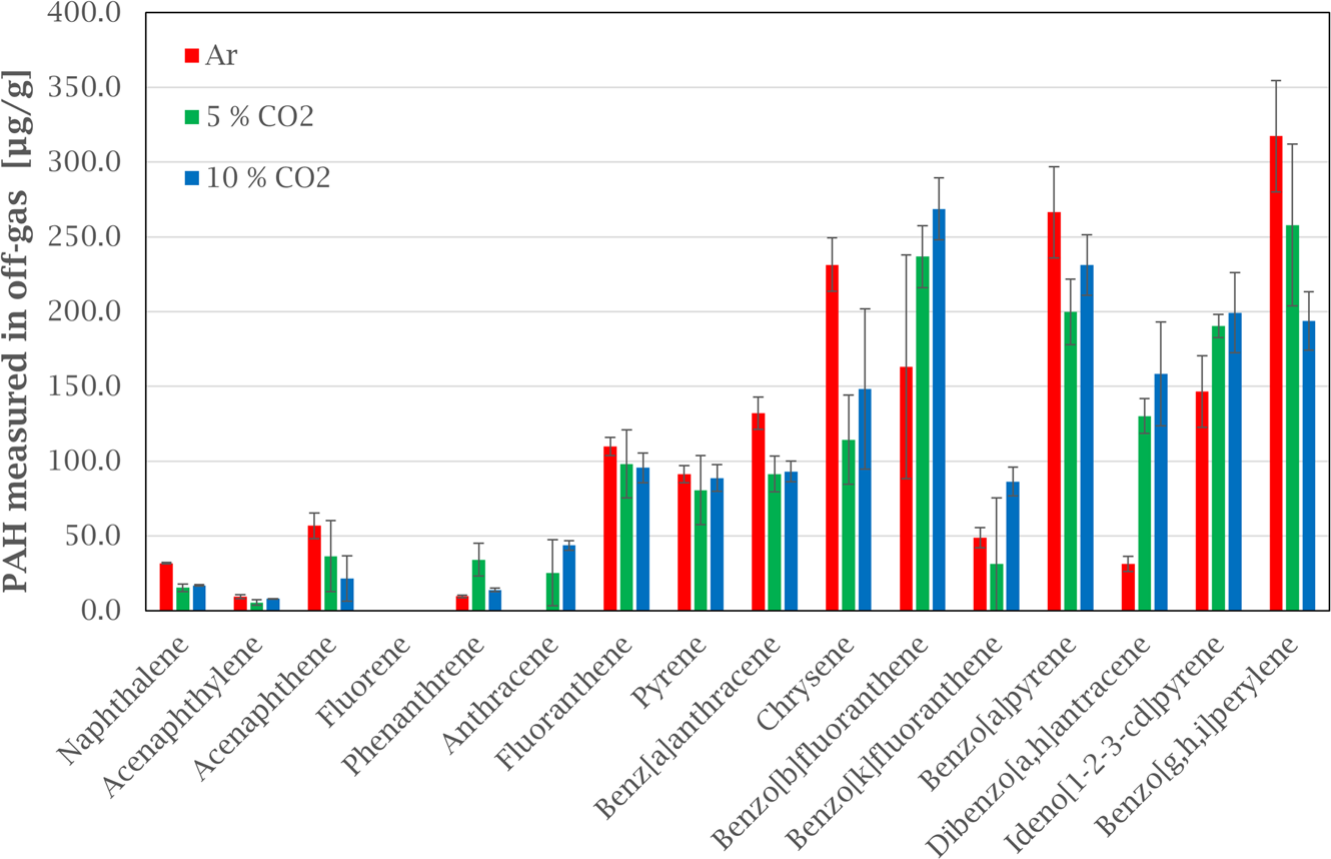
Comparing EPA-16 PAH emissions from green anode paste heated in argon and CO_2_ (5 and 10%) atmospheres. Error bars show the standard deviation for each compound based on three experiments.

The effect of the atmosphere on the PAH emissions is shown in more detail in Figures 6 and 7. Compared to inert atmosphere, adding 5% oxygen reduced the total PAH concentration by 65%, from 1645 μg/g to 569 μg/g. At 10% oxygen, the total PAH concentration was reduced with 75%, to 417 μg/g (Figure 6). This, seen together with the increased mass loss for the GAP sample (Figure 3), with increasing oxygen levels, indicate increased combustion of the anode paste.

With the temperatures and residence time of the experiment, 10% O_2_ is not enough to achieve complete combustion of PAH emitted from GAP. High molecular weight PAHs seem to be more affected by the oxygen level than lower molecular weight PAHs, as the concentration level of four ring PAHs (fluoranthene to chrysene in Figure 6) does not change significantly when the oxygen level increase. As described by Sun^13^ and Liu et al.,^14^ complete combustion of PAH depends on several factors such as temperature and oxygen levels. In this study, the seemingly stable level of four ring PAHs could be the result of combustion products from the decomposition of other HMW PAH compounds, where the insufficient temperature levels and available oxygen could lead to stabilization of the radical combustion products. The majority of the emissions from the experiments occur at temperatures of 500 °C or lower (Figure 4), where the HMW PAHs would be in a condensed state. In this state, the molecules could be susceptible to catalytic degradation in a gas–solid phase initiated by the trace elements nickel, vanadium, and iron in the GAP mixture, as described by Pujro et al.^15^

Adding CO_2_ to the atmosphere did not affect the total PAH concentration significantly compared to the argon atmosphere. The total PAH concentration for atmospheres with 5 and 10% CO_2_ were 1547 and 1666 μg/g, respectively (Figure 7).

The weight loss of GAP samples in CO_2_ was similar to that in argon, indicating that no significant oxidation of the material occurred. This finding is contradictory to that of Chevarin et al.^8^ However, in the current study, the maximum temperature was 750 °C as opposed to 960 °C, and the differences in temperature would influence the CO_2_ reactivity of the anode materials; in addition the change in the levels of PAH emitted at temperatures above 500 °C could be too low for an effect to be statistically significant.

### Breakthrough Test

Overall, the amount of LMW PAHs present was low for all experiments compared to the HMW species. To confirm this, a repetition of the experiment in an argon atmosphere was therefore performed to test for breakthrough of LMW PAHs from the 2-propanol in the sample collection system. It was done by connecting thermal desorption tubes with absorbent media at the end of the sampling line. Table 2 shows the results of PAHs in the tubes, compared to the average of three experiments of PAHs in 2-propanol.

**Table 2:** Concentration of PAH Species Found in Tubes and 2-Propanol after Experiment with GAP in Argon Atmosphere^a^

PAH species	tube [μg/g]	2-propanol [μg/g]
naphthalene	0.2	31.7
acenaphthene	5.2	56.7
phenanthrene	10.0	9.5
anthracene	2.3	0.0
fluoranthene	22.9	109.7

a Values in 2-propanol are an average of three experiments, and results from tubes are from a single experiment.

Results show low amounts of the smallest PAHs (naphthalene and acenaphthene) in the tubes. Fluoranthene is detected in the greatest concentration in the tube out of the five PAHs, corresponding to the relative amounts in the 2-propanol bottles. The noticeable amount of fluoranthene in the tubes could hence be explained by evaporation of 2-propanol, containing PAHs, from sampling bottles to the tube, if the solvent was not kept at sufficiently low temperatures during the experiment. If this occurred, all species present in 2-propanol with lower boiling points than fluoranthene would also be transferred to the tube, as illustrated by data in Table 2.

### Pyrolysis GC-MS

**Figure 8:**
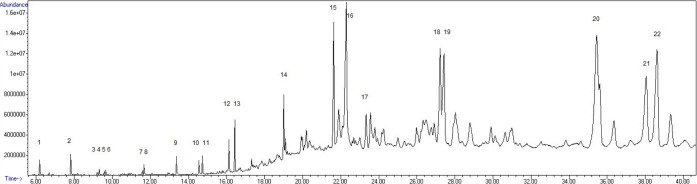
Spectrum showing signals from pyrolysis GC-MS of GAP at 750 °C. Abundance is shown on the *y*-axis and time on the *x*-axis.

Pyrolysis of GAP at 400 and 600 °C resulted in similar pyrolytic profiles, both with few identifiable signals. Pyrolysis at 750 °C produced a species profile presented in Figure 8, corresponding to the identified species in Table 3.

**Table 3:** Detected and Identified PAH Species from Pyrolysis GC-MS of GAP at 750 °C

ID no.	species
1	Benzene
2	Toluene
3	Xylene
4	Xylene
5	Styrene
6	Xylene
7	Indane
8	Indane
9	Naphthalene
10	Naphthalene, 1-methyl-
11	Naphthalene, 2-methyl-
12	Biphenylene
13	Acenaphalene
14	Antracene/Phenanthrene
15	Fluoranthene/Pyrene
16	Aromatic, Not identified
17	Pyrene, 1-methyl-
18	Tripenylen/Benz(a)anthracene
19	Tripenylen/Benz(a)anthracene
20	Benzo(e)pyrene/Benzo(k)fluoranthene
21	Benzo(e)pyrene/Perylene/Benzo(a)pyrene
22	Benzo(e)pyrene/Perylene/Benzo(a)pyrene

Isomer PAH can not be identified as references for these compounds were not analyzed. Components are not quantified using this technique, but looking at signal abundance and area, a similar trend is shown when comparing with results from 2-propanol samples. The strongest signals originate from components antracene/phenanthrene (signal no. 14, Figure 8) and larger MW PAHs. Together, this confirms the low concentration of LMW PAHs in the 2-propanol samples, and in the tube samples, originating from the green anode paste.

Samples pyrolyzed in the alumina tube furnace produced PAH emissions in the temperature range of 400 to 600 °C (Figure 4), in contrast to the pyrolysis GC-MS experiments, and the reason behind the varying results from the two techniques could be the time of heating for the sample, where the pyrolysis GC-MS use 1 min, while a tube furnace experiment lasts several hours (Figure 2b).

## Conclusions

The effects of temperature and atmosphere on EPA-16 PAH emissions from green anode paste were investigated in a laboratory alumina tube furnace setup with off-line PAH analysis. Atmospheres tested were argon, CO_2_, and O_2_ with the temperature interval ranging from room temperature to 750 °C. Results show PAH emissions at the level of 1645 μg/g, and 1547 and 1666 μg/g, from inert and CO_2_ atmospheres (5% and 10%). With added oxygen, concentrations decreased to 569 μg/g and 417 μg/g for 5% and 10% O_2_, respectively, corresponding to a 65% and 75% decrease in emission. For the current setup with the given temperatures, available oxygen and species resident time, complete combustion of PAHs was not achieved. A suggestion for future work is expanding the analysis techniques for the off-gas to include gas components such as CO/CO_2_ and H_2_ and other VOCs to investigate the formation and destruction mechanisms of PAHs in more detail. PAHs are persistent molecules, and sufficient levels of oxygen, temperature, and residence time are needed to achieve complete combustion.
